# Membrane Properties of Striatal Direct and Indirect Pathway Neurons in Mouse and Rat Slices and Their Modulation by Dopamine

**DOI:** 10.1371/journal.pone.0057054

**Published:** 2013-03-01

**Authors:** Henrike Planert, Thomas K. Berger, Gilad Silberberg

**Affiliations:** 1 Department of Neuroscience, Karolinska Institute, Stockholm, Sweden; 2 Cluster of Excellence NeuroCure and Department of Experimental Neurology, University Medicine Charité, Berlin, Germany; 3 Department of Molecular and Cell Biology, University of California, Berkeley, United States of America; University of Bristol, United Kingdom

## Abstract

D1 and D2 receptor expressing striatal medium spiny neurons (MSNs) are ascribed to striatonigral (“direct”) and striatopallidal (“indirect”) pathways, respectively, that are believed to function antagonistically in motor control. Glutamatergic synaptic transmission onto the two types is differentially affected by Dopamine (DA), however, less is known about the effects on MSN intrinsic electrical properties. Using patch clamp recordings, we comprehensively characterized the two pathways in rats and mice, and investigated their DA modulation. We identified the direct pathway by retrograde labeling in rats, and in mice we used transgenic animals in which EGFP is expressed in D1 MSNs. MSNs were subjected to a series of current injections to pinpoint differences between the populations, and in mice also following bath application of DA. In both animal models, most electrical properties were similar, however, membrane excitability as measured by step and ramp current injections consistently differed, with direct pathway MSNs being less excitable than their counterparts. DA had opposite effects on excitability of D1 and D2 MSNs, counteracting the initial differences. Pronounced changes in AP shape were seen in D2 MSNs. In direct pathway MSNs, excitability increased across experimental conditions and parameters, and also when applying DA or the D1 agonist SKF-81297 in presence of blockers of cholinergic, GABAergic, and glutamatergic receptors. Thus, DA induced changes in excitability were D1 R mediated and intrinsic to direct pathway MSNs, and not a secondary network effect of altered synaptic transmission. DAergic modulation of intrinsic properties therefore acts in a synergistic manner with previously reported effects of DA on afferent synaptic transmission and dendritic processing, supporting the antagonistic model for direct vs. indirect striatal pathway function.

## Introduction

Medium spiny neurons (MSNs) form the vast majority of striatal neurons and project *directly* or *indirectly*, via the external globus pallidus (GPe), to BG output structures substantia nigra *pars reticulata* (SNr) and internal globus pallidus (GPi). These projections have been the basis of a functional model, where the direct striatonigral and striato-GPi pathway facilitates and the indirect striato-GPe pathway inhibits movements [1]. However, MSNs of both pathways share many morphological and electrophysiological properties, as well as synaptic inputs [2]–[4]. Earlier slice studies on general electrophysiological properties of MSNs were mainly performed in rats, but studies addressing intrinsic properties of the two MSN types were exclusively done in transgenic mice [5]–[7]. There has been, however, substantial discussion in the past about the specificity of D1 and D2 receptor (D1 R/D2 R) expression for direct and indirect pathway MSNs, respectively [8]. We therefore identified direct pathway MSNs with two different methods, and in two different species: In the rat, using retrograde labeling of SNr projecting MSNs with fluorescent latex beads, and in BAC Drd1a-EGFP mice. In order to unravel differences in intrinsic electrical properties, we used a detailed stimulation protocol that captures a wide range of passive and active membrane properties.

Dopamine (DA) has long been proposed to lead to differential effects on the striatal projection systems [1], based on evidence for the opposite effect of DA depletion on activity of the pathways. Within this framework, DA should increase direct pathway excitability and decrease indirect pathway excitability. At the synaptic level, DA affects glutamate release, as well as NMDA and AMPA currents in such opposite ways, depending on DA R expression [9]. However, the net effects of DA and selective receptor agonists on intrinsic MSN excitability have not been easy to elucidate [10]. Most studies have been done on dissociated and partly identified MSNs [11]–[14], or investigating the effect of various DA R agonists and antagonists on unselected MSNs [13]–[15]. The direct impact of DA on MSNs of the two projection systems within the intact striatal microcircuit is, however, still unclear.

In this study, we quantified passive and active membrane properties of direct pathway MSNs and compared them with the respective nonlabeled (putative indirect pathway) population, using two different methods of identification in two different species. To investigate the direct effect of DA on MSNs of both types, we bath-applied DA and recorded from identified MSNs.

While most electrical properties were similar, a difference in membrane excitability was apparent across species, in which direct pathway MSNs were less excitable than indirect pathway MSNs. We provide evidence that, in mice, DA increases intrinsic excitability in D1 (direct pathway) MSNs and reduces excitability in D2 (indirect pathway) MSNs, thus counteracting differences seen under control conditions. Excitability increases were direct and D1 R mediated in direct pathway MSNs.

## Results

**Figure 1:**
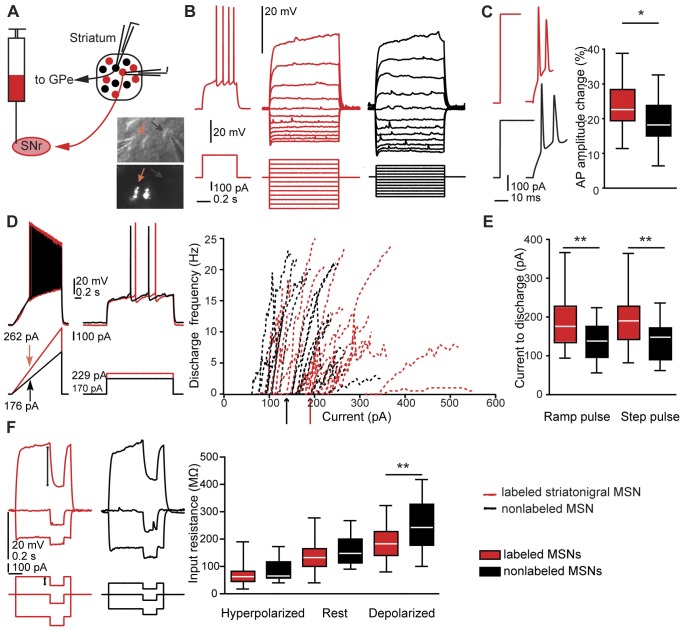
Membrane properties of MSN subtypes in rat slices. *A*, schematic figure of retrograde labeling of striatonigral medium spiny neurons (MSNs), and patch-clamp recording from direct pathway (red) and nearby nonlabeled (putative indirect pathway) MSNs (black). *A*, lower right inset, example of nearby patched labeled and nonlabeled MSN (red and black arrows, respectively). Upper panel: IR-DIC image. Lower panel: epifluorescence image of the same neurons. *B*, left, typical delayed discharge pattern of a retrogradely labeled striatonigral neuron (MSN). *B*, middle and right, current-voltage relationship of a labeled (red) and a nonlabeled MSN (black). The same color code is used throughout Figure 1. *C*, left, typical APs in response to strong step currents. *C,* right, relative AP amplitude change between first and second AP. The amplitude decreased to a larger extent in striatonigral than in nonlabeled MSNs (n = 26 and n = 25, p = 0.014, t-test). *D*, left, voltage responses to ramp currents injected into labeled and nonlabeled MSNs. *D*, middle, minimal suprathreshold step currents (rheobase). *D*, right, current-frequency relationship of individual MSNs and average step current for minimal discharge for the two MSN populations (arrows). *E*, box plot showing differences in minimal ramp and step currents leading to discharge. MSNs projecting to substantia nigra were less excitable by both current ramps and steps (ramps, n = 26 and n = 23, p<0.01; steps, n = 25 and n = 21, p<0.01; t-tests). *F*, left, stimulation protocol and response of individual MSNs used to calculate input resistance at different membrane potentials. *F*, right, box plot showing input resistance for the two MSN populations. Input resistance was significantly different at depolarized membrane potentials (labeled neurons, n = 25; nonlabeled neurons, n = 22; p = 0.008, t-test). Single-cell traces in *B, C, D* and *F* are from the same two MSNs.

**Figure 2:**
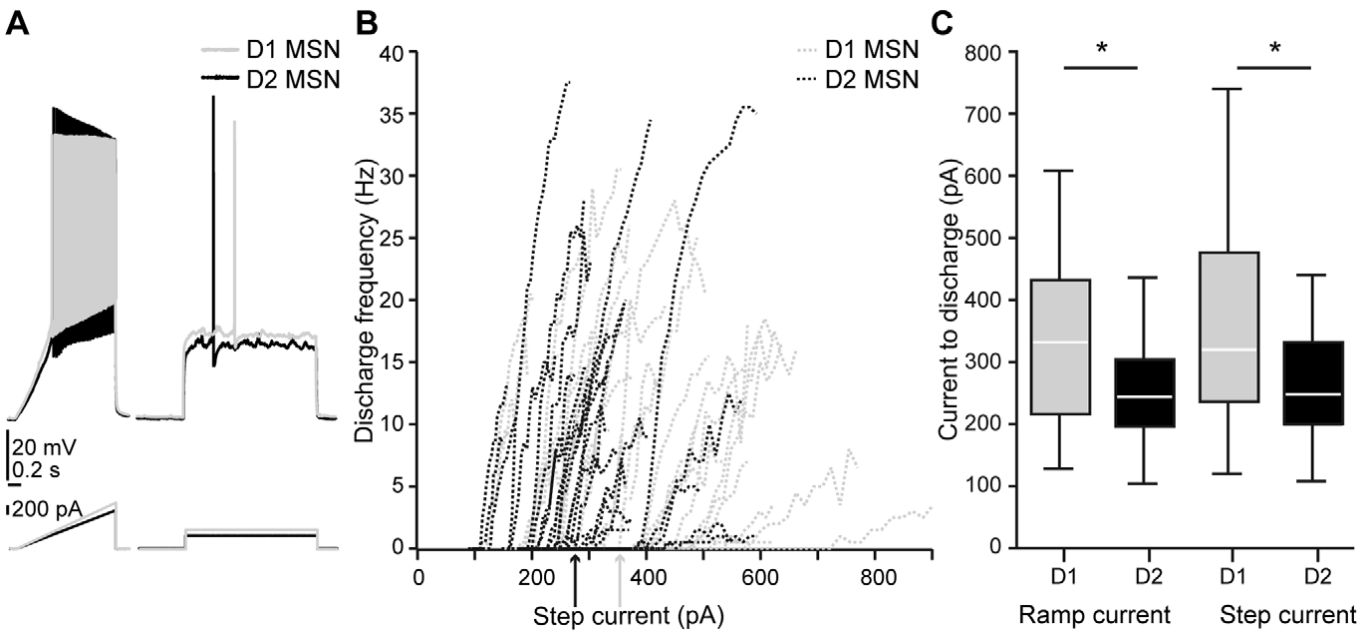
Differential excitability of mouse MSNs. *A*, voltage response of Drd1-EGFP expressing D1 MSN (gray) and nonlabeled D2 MSN (black) to ramp and step current injection. *B*, current-frequency relationship of individual D1 and D2 MSNs (dotted lines) and average step current to threshold discharge for the two MSN populations (arrows). *C*, D1 MSNs were less excitable than D2 MSNs. This was seen in the minimal current for threshold discharge, both for ramp and step currents (n = 31/29 and 30/28, respectively, p<0.05, t-tests).

**Figure 3:**
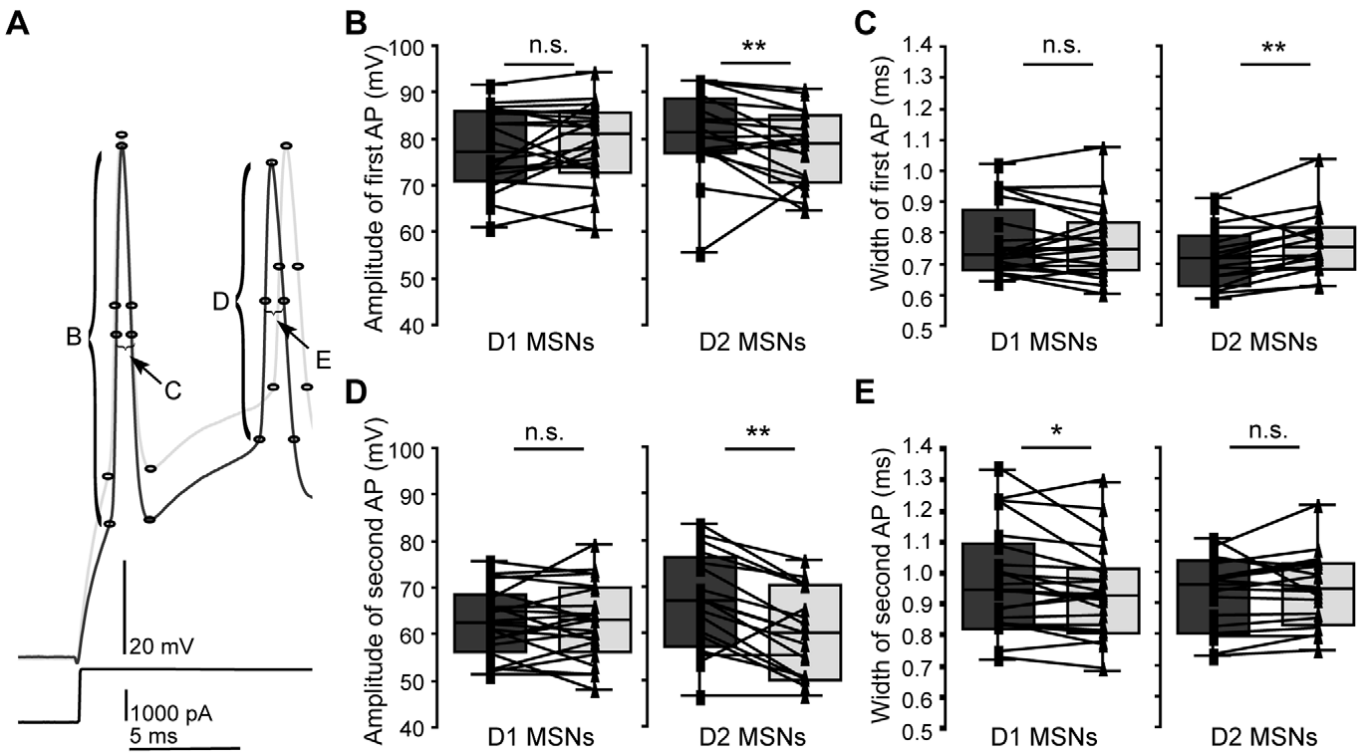
Changes in AP properties after DA application in D1 and D2 MSNs. APs were smaller and wider after DA application in D2 MSNs. *A,* example traces of two consecutive APs in response to step current injection before and after application of DA (60 µM, dark and light gray, respectively). *B, C,* box plots, changes in amplitude (*B*) and width (*C*) of the first AP after application of DA in D1 and D2 MSNs (left and right panels, respectively). *B, C*, left panels, no significant changes were observed in D1 MSNs. *B, C*, right panels, in D2 MSNs, however, the amplitude of the first AP decreased (p = 0.044) and AP width increased (p = 0.002). *D, E,* box plots, amplitude and width of the second AP before and after application of DA. *D,* left panel, the amplitude of the second AP did not change significantly in D1 MSNs (p = 0.929). *D,* right panel, in D2 MSNs, the amplitude again strongly decreased (p<0.001). *E,* left and right panels, width of the second AP decreased in D1 MSNs (p = 0.032), but did not change significantly in D2 MSNs (p = 0.439). See also Tables 3 and 4.

We obtained patch clamp recordings from MSNs in rat and mouse striatum in which direct pathway striatonigral or D1 MSNs were fluorescently marked by retrograde labeling and EGFP, respectively (see **Materials and Methods**). Recorded MSNs of the different output systems were held at hyperpolarized baseline membrane potential (near −80 mV). We then measured, with a series of step and ramp current injection protocols, various aspects of the voltage response (see Figs. 1, 2, 3). We extracted general passive properties such as input resistances and membrane time constants at different membrane potentials, as well as excitability measures (discharge threshold, minimal step and ramp currents needed to obtain threshold discharge). We also describe action potential (AP) properties such as width and amplitude of consecutive APs in a train.

### Differential Membrane Properties of Striatal Projection MSNs in Two Model Systems

Comparing the pooled data of both MSN types between species revealed that mouse MSNs were less excitable than rat MSNs, which was seen both in the step and ramp currents needed to obtain threshold discharge: The minimal step current leading to discharge was 167.54±64.9 pA in rats vs. 314.6±134.2 pA in mice, respectively (n = 46 and 59, p<0.00001). Minimal ramp current for discharge was 163.9±59.2 pA and 296.5±115.3 pA (p<0.00001). This difference may be partly due to the fact that the baseline membrane potential from which the stimulation was administered was more depolarized for rat MSNs (−75.22±6.20 mV vs. −79.32±2.57 mV, p = 0.00002), but also input resistances, as well as the time constants measured at different membrane potentials were substantially lower in mice (e.g. input resistance at rest 150.76±58.32 MΩ in rats and 90.56±48.75 MΩ in mice, p<0.00001, time constant 11.07±3.84 ms in rats and 5.35±2.96 ms in mice (p<0.00001)). A likely reason for this difference is that mouse slices were obtained from older animals (PN 21 to 32) than rat slices (PN 14–19), as it has been shown that neuronal input resistance, membrane time constant, as well as excitability decrease with age [16]. Recordings from juvenile d15 mouse MSNs confirmed this assumption, as in these excitability was higher than that from the older mice as measured by both step and ramp currents (192±26 pA and 221±32 pA, n = 9 and 10, p = 0.008 and 0.04, respectively). Excitability as measured by step currents in young mice was however similar to that of juvenile rats (p = 0.28).

### Direct Pathway MSNs are Less Excitable Across Species

Before transgenic mice were widely available, the vast majority of striatal slice studies were performed in rats, and it is a matter of debate whether in rats D1 and D2 R expressing neurons correspond to direct vs indirect pathways, respectively (see **Introduction**). The recent studies comparing neuronal populations have however been performed in the mouse model. Here, we identified the direct pathway by retrograde labeling in the rat, thereby bridging a gap between studies in unselected MSNs in the rat slice preparation and findings in the mouse. To our knowledge, this is the first time that electrophysiological parameters of the different neuron types are extensively analyzed in the rat, and they are also for the first time directly related to data from transgenic mice. Similarities and differences between membrane properties in rat labeled direct pathway and nonlabeled MSNs are summarized in Table 1. The resting membrane potentials were similar (p = 0.435), as was the AP threshold (p = 0.655). When eliciting APs with step current pulses, we did not observe differences between the MSN types in average amplitude, duration or width of the first or second AP in a train. The AP amplitude reduction between the first and the second AP was larger in striatonigral MSNs (p = 0.014, Fig. 1*C*). This was rather due to a decreased rising rate of the second AP (p = 0.037), than to differential changes in fall-rate or duration (fall-rate change p = 0.978; duration change p = 0.392). However, despite similar AP thresholds, striatonigral MSNs were less excitable than nonlabeled MSNs, as seen in the ramp current leading to the first AP, as well as the average minimal step current needed to reach threshold discharge (p = 0.005, Fig. 1*D, E*). These differences were paralleled by different input resistances at membrane potentials depolarized from rest (p = 0.008, Fig. 1*F*). Also, the membrane time constant for a brief (5 ms) hyperpolarizing pulse (delta pulse) was significantly shorter in striatonigral as compared to nonlabeled MSNs (p<0.001), and the same was true for the membrane time constant measured at depolarized membrane potential.

**Table 1:** Membrane properties of rat MSN subtypes.

	Striatonigral MSNs (n = 32 )			Nonlabeled MSNs (n = 26)			
	n	mean	SD	n	mean	SD	p-value
Rest potential when impaling cell (mV)	29	−67.67	12.13	26	−64.87	14.25	0.435
Baseline current (pA)	26	−25.40	59.89	24	−5.64	24.09	0.130
Baseline membrane potential for recordings (mV)	32	−74.97	6.49	25	−75.55	5.93	0.731
Amplitude of first AP (mV)	32	82.23	10.03	26	82.55	8.26	0.898
Duration of first AP (ms)	32	3.11	0.49	26	2.99	0.37	0.322
Width of first AP (ms)	32	1.16	0.20	26	1.10	0.11	0.122
Amplitude of second AP (mV)	26	62.72	9.74	25	66.61	6.82	0.106
Duration of second AP (ms)	25	3.58	0.80	25	3.60	0.76	0.941
Width of second AP (ms)	26	1.62	0.35	25	1.54	0.22	0.346
**Amplitude change first to second AP (%)**	26	−23.90	6.80	25	−19.20	6.39	**0.014**
AP duration change (%)	25	15.72	17.09	25	19.62	14.67	0.392
AP width change (%)	26	40.14	16.87	25	40.73	56.50	0.887
Input resistance at rest (MΩ)	25	138.39	57.35	23	164.20	57.59	0.127
**Input resistance [depolarized] (MΩ)**	25	189.57	61.70	22	252.14	91.23	**0.008**
Input resistance [hyperpolarized] (MΩ)	25	73.71	42.74	23	86.80	40.51	0.283
Membrane time constant at rest (ms)	29	12.98	8.83	25	13.85	6.25	0.684
**Membrane time constant [depolarized] (ms)**	31	18.54	6.33	25	26.28	9.18	**0.000**
Membrane time constant [hyperpolarized] (ms)	31	4.31	4.31	26	6.17	4.46	0.075
**Membrane time constant for delta pulse (ms)**	32	9.50	3.54	25	13.08	3.27	**0.000**
Threshold for APs (mV)	32	−42.42	4.57	26	−41.94	3.19	0.655
AP drop rate (mV/pA)	26	−0.07	0.04	23	−0.08	0.05	0.269
**Ramp current to first spike (pA)**	26	185.86	62.68	23	139.15	44.36	**0.005**
**Minimum step current for discharge (pA)**	25	191.42	67.36	21	139.10	49.58	**0.005**
Slope of current-frequency relationship (Hz/pA)	25	0.16	0.12	21	0.21	0.11	0.196

In mice, D1 and D2 MSNs were remarkably similar in their membrane properties (see Table 2). They displayed similar resting membrane potentials shortly after transition to whole-cell recording mode (p = 0.834). The AP thresholds were −42.73±2.65 mV and −44.29±4.44 mV for D1 and D2 MSNs, respectively, and the difference failed to reach significance (p = 0.112). D1 MSNs were however less excitable than D2 MSNs in terms of current needed to obtain discharge (p = 0.010, Fig. 2).

**Table 2:** Membrane properties of mouse D1 and D2 MSNs.

	D1 MSNs (n = 32)			D2 MSNs (n = 30)			
	n	mean	SD	n	mean	SD	p-value
Rest potential when impaling cell (mV)	24	−78.65	4.75	25	−78.91	3.93	0.834
Baseline current (pA)	30	−4.13	47.17	29	−6.25	73.16	0.895
Baseline membrane potential for recordings (mV)	32	−78.97	2.15	29	−79.70	2.97	0.266
Amplitude of first AP (mV)	29	78.50	7.73	29	78.77	9.42	0.907
Duration of first AP (ms)	29	1.83	0.42	29	1.75	0.49	0.523
Width of first AP (ms)	29	0.77	0.10	29	0.75	0.11	0.352
Amplitude of second AP (mV)	29	62.16	8.29	29	64.34	10.04	0.371
Duration of second AP (ms)	29	1.94	0.35	29	1.92	0.33	0.803
Width of second AP (ms)	29	1.00	0.18	29	0.97	0.15	0.472
Amplitude change first to second AP (%)	29	−20.75	7.90	29	−18.18	8.81	0.247
AP duration change (%)	29	7.34	11.70	29	12.40	14.55	0.150
AP width change (%)	29	29.42	13.55	29	30.28	9.80	0.782
Input resistance at rest (MΩ)	30	85.26	48.77	27	96.44	48.97	0.392
Input resistance [depolarized] (MΩ )	31	131.70	57.79	28	151.37	71.51	0.248
Input resistance [hyperpolarized] (MΩ )	31	50.67	29.46	28	59.90	28.87	0.230
Membrane time constant at rest (ms)	29	5.16	2.39	28	5.98	3.00	0.254
Membrane time constant [depolarized] (ms)	32	11.05	3.15	29	12.43	4.37	0.159
Membrane time constant [hyperpolarized] (ms)	31	1.84	1.08	29	2.54	2.11	0.114
Membrane time constant for delta pulse (ms)	30	4.84	2.43	29	5.87	3.38	0.186
Threshold for APs (mV)	32	−42.73	2.65	28	−44.29	4.44	0.112
AP drop rate (%)	30	−0.02	0.02	28	−0.02	0.02	0.668
**Ramp current to first spike (pA)**	31	332.93	127.30	29	257.53	87.11	**0.010**
**Minimum step current for discharge (pA)**	30	355.39	150.33	28	268.74	96.07	**0.011**
Slope of current/frequency relationship (Hz/pA)	30	0.12	0.08	27	0.15	0.09	0.174

The difference in excitability between the two MSN subtypes was the common feature we observed in both mice and rats, such that direct pathway MSNs were less excitable than their counterparts.

### Dopamine Depolarizes Direct Pathway MSNs

We next studied the impact of DA on membrane potential and intrinsic properties of the two MSN subtypes, especially on passive and active properties underlying excitability. We focused on mice for DA modulation experiments for several reasons: Firstly, effects of DA and agonists on excitability in rat slice studies have been very hard to interpret [10]. In the mouse model on the other hand, clearly different DAergic effects on the two MSN populations on synaptic (and dendritic) excitability were seen [9], [17]. We were interested in investigating whether these changes were paralleled by changes in whole-cell excitability. Secondly, specificity for the D1 R expressing cells in Drd-BAC mice for the direct pathway has been demonstrated [5], [18], so that we can assume that the vast majority of labeled cells are D1 R expressing as well as direct pathway projecting. This remains to be shown for rats.

When bath-applying DA (60 µM) at depolarized membrane potentials near −60 mV, MSNs showed an overall depolarizing response (n = 21, Δ membrane potential = 4.64±7.30 mV, p<0.001, two-way ANOVA, data not shown). However, Bonferroni post-hoc tests revealed a significant change for D1 MSNs only (6.99 mV ±7.30, n = 10, p<0.01). Both the repeated step currents and the DA application may have induced this effect. We therefore repeated the protocol in the absence of DA. In this case, no significant changes in membrane potential overall or in the individual MSN types were observed (n = 4 and 5 for D1 and D2 MSNs, respectively, p = 0.456 for main effect of time, two-way ANOVA, and p>0.05 in Bonferroni post-hoc tests, data not shown), suggesting that the changes in membrane potential were mediated by DA. When applying DA at hyperpolarized membrane potential near −80 mV in 14 D1 MSNs and 6 D2 MSNs, no consistent effect on membrane potential was observed (p>0.05 two-way ANOVA and Bonferroni post-hoc tests).

### DA Affects Different AP Properties in D1 and D2 MSNs

We investigated the effect of DA on the detailed membrane properties of recorded MSNs (26 D1 MSNs and 19 D2 MSNs). Table 3 and Table 4 show the extracted parameters in D1 and D2 MSNs, respectively. Consistent with the depolarizing responses of MSNs described above, less current was required to hold both D1 and D2 MSNs at membrane potentials near −80 mV following DA application (p = 0.000 and p = 0.006, respectively). In both MSN types, AP properties changed with the application of DA (Fig. 3, Tables 3 and 4). In D1 MSNs, the width of the first AP in a train did not change (p = 0.659, Fig. 3*C*), but the second AP became shorter (p = 0.032, 3*E*). In D2 MSNs, the effects of DA on AP properties were more pronounced (Fig. 3*B, C, D*, Table 4). Following DA application, the amplitude of the first AP in a train decreased (p = 0.044, Fig. 3*B*), and both duration and width of the first AP increased (p = 0.004 and 0.002, respectively, Table 4, Fig. 3*C*). DA also strongly decreased the absolute amplitude of the second AP in a train (p<0.001, Fig. 3*D*). In summary, APs became smaller and wider after DA application in D2, but not D1 MSNs.

**Table 3:** Effect of dopamine (60 µM) on membrane properties of D1 MSNs.

	Control		DA			
	mean	SD	mean	SD	n	p-value
**Baseline current (pA)**	−5.10	47.03	−35.19	58.08	25	**0.000**
Baseline membrane potential for recordings (mV)	−79.18	2.28	−79.13	3.13	25	0.901
Amplitude of first AP (mV)	78.04	8.36	79.53	8.16	22	0.230
Duration of first AP (ms)	1.84	0.46	1.89	0.58	22	0.352
Width of first AP (ms)	0.77	0.11	0.77	0.11	22	0.659
Amplitude of second AP (mV)	62.74	7.29	62.83	8.20	22	0.929
Duration of second AP (ms)	1.90	0.34	1.86	0.34	22	0.132
**Width of second AP (ms)**	0.98	0.17	0.94	0.15	22	**0.032**
Amplitude change first to second AP (%)	−19.47	6.03	−20.99	6.22	22	0.145
**AP duration change (%)**	5.41	11.91	1.29	12.91	22	**0.015**
**AP width change (%)**	26.44	11.16	22.96	9.31	22	**0.010**
Input resistance at rest (MΩ )	89.40	53.64	88.86	52.90	22	0.912
Input resistance [depolarized] (MΩ )	132.40	59.38	140.90	73.61	22	0.168
Input resistance [hyperpolarized] (MΩ )	52.80	32.36	56.66	37.63	23	0.171
Membrane time constant at rest (ms)	5.20	2.62	4.95	2.31	22	0.334
Membrane time constant [depolarized] (ms)	11.10	3.32	11.79	5.25	24	0.362
**Membrane time constant [hyperpolarized] (ms)**	1.91	1.20	2.23	1.85	24	**0.048**
Membrane time constant for delta pulse (ms)	4.87	2.70	5.02	2.61	23	0.383
**Threshold for APs (mV)**	−42.36	2.58	−44.02	3.64	25	**0.050**
**AP drop rate (mV/pA)**	−0.02	0.03	−0.03	0.03	21	**0.004**
**Ramp current to first spike (pA)**	329.00	126.80	311.30	122.60	25	**0.017**
**Minimum step current for threshold discharge (pA)**	334.90	150.10	315.20	139.10	21	**0.020**
Slope of current/frequency relationship (Hz/pA)	0.14	0.07	0.12	0.08	21	0.132

**Table 4:** Effect of dopamine (60 µM) on membrane properties of D2 MSNs.

	Control		DA			
	mean	SD	mean	SD	n	p-value
**Baseline current (pA)**	−4.87	67.20	−25.09	63.24	19	**0.006**
Baseline membrane potential for recordings (mV)	−79.30	2.95	−78.84	2.39	18	0.410
**Amplitude of first AP (mV)**	81.45	9.08	78.10	7.77	18	**0.044**
**Duration of first AP (ms)**	1.66	0.36	1.81	0.39	18	**0.004**
**Width of first AP (ms)**	0.72	0.09	0.77	0.10	18	**0.002**
**Amplitude of second AP (mV)**	66.89	10.43	60.26	9.07	18	**0.000**
Duration of second AP (ms)	1.85	0.26	1.81	0.23	18	0.110
Width of second AP (ms)	0.93	0.12	0.94	0.12	18	0.439
**Amplitude change first to second AP (%)**	−17.86	8.57	−22.76	9.26	18	**0.000**
**AP duration change (%)**	13.29	11.92	1.99	12.11	18	**0.000**
**AP width change (%)**	29.71	8.89	23.28	6.45	18	**0.000**
**Input resistance at rest (MΩ )**	86.83	41.30	93.97	43.04	17	**0.020**
Input resistance [depolarized] (MΩ )	134.70	46.60	135.90	41.31	17	0.770
**Input resistance [hyperpolarized] (MΩ )**	56.06	25.57	68.54	32.29	18	**0.000**
Membrane time constant at rest (ms)	5.28	2.14	5.11	2.41	18	0.467
Membrane time constant [depolarized] (ms)	11.19	2.93	10.97	3.56	18	0.689
Membrane time constant [hyperpolarized] (ms)	2.58	2.51	3.68	4.70	19	0.261
Membrane time constant for delta pulse (ms)	5.55	3.51	5.29	3.45	19	0.096
**Threshold for APs (mV)**	−45.37	4.25	−43.11	4.49	18	**0.000**
AP drop rate (mV/pA)	−0.03	0.02	−0.02	0.02	18	0.244
Ramp current to first spike (pA)	263.70	84.65	272.10	76.55	19	0.322
Minimum step current for discharge (pA)	246.90	77.12	264.90	82.51	15	0.061
**Slope of current/frequency relationship (Hz/pA)**	0.17	0.09	0.13	0.09	15	**0.001**

### Dopamine Modulates Excitability in D1 and D2 MSNs in Opposite Directions

In studies investigating the effect of D1 and D2 R agonists and antagonists on unselected MSNs, D1 R stimulation increased evoked discharge via modulation of L-type Ca^2+^ channels at depolarized membrane potentials near −55 mV [15]. These channels are negatively modulated via D2 mechanisms, complementing the suppression of evoked activity by D2 agonists [13]. We hypothesized that directly applying DA to the network would lead to increased discharge in D1 MSNs and decreased discharge in D2 MSNs at relatively depolarized membrane potential [10], [19].

**Figure 4:**
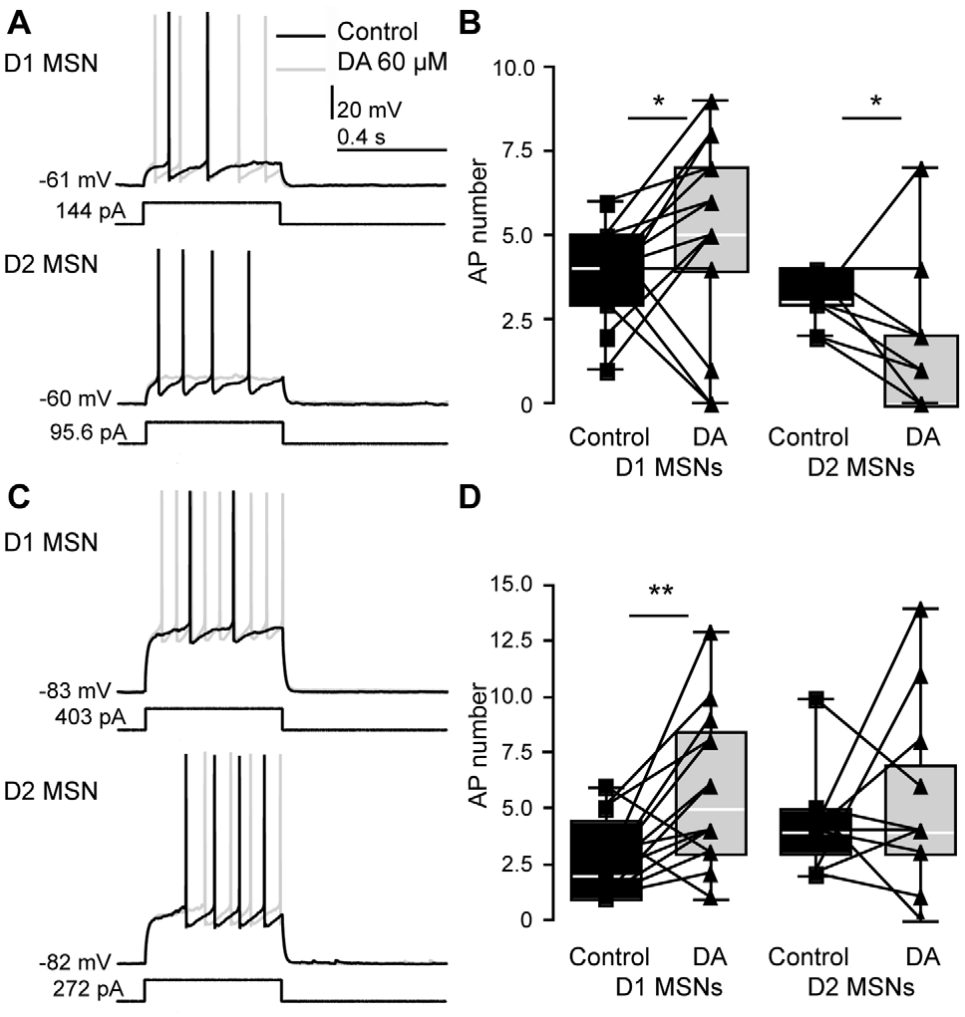
Effects of DA on excitability of MSN subtypes at depolarized and hyperpolarized membrane potential. *A*, voltage response of D1 and D2 MSNs at depolarized membrane potentials in response to suprathreshold step currents before and after application of DA (60 µM, dark and light gray traces, respectively), demonstrating differential modulation of discharge in the different MSN types. *B*, box plots showing the effect of DA on excitability near −60 mV in D1 and D2 MSNs (−60.84±1.76 mV and −62.69±1.66 mV, n = 15 and n = 11, respectively). Discharge increased in D1 MSNs and decreased in D2 MSNs (D1 MSNs, number of APs before DA application: 3.67±1.45, after DA: 5.07±2.81, n = 15, p = 0.046; D2 MSNs: number of APs before application 3.27±0.79, after DA application 1.91±2.07; n = 11, p = 0.031, one-tailed paired t-tests). *C, D*, when the effect of DA was tested near −80 mV (−81.71±2.21 mV, n = 26), D1 MSNs increased spiking in response to the same step current (number of APs before DA application: 2.63±1.69, after application: 5.78±3.42; n = 14, p = 0.0075, two-tailed paired t-test), whereas D2 MSNs showed no change in discharge (number of APs before DA application: 4.25±2.14, after DA application: 5.17±4.04, n = 12, p = 0.508 two-tailed paired t-test).

We studied neuronal discharge during injection of identical current steps given at two different membrane potentials (either −60 mV or −80 mV). Indeed, near −60 mV, neuronal excitability increased in D1 MSNs and decreased in D2 MSNs (n = 15, p = 0.046, and n = 11, p = 0.031, respectively, Fig. 4*A, B*). At hyperpolarized membrane potentials, modulation of other conductances may be more relevant for the net effect of DA, and D1 R stimulation has been proposed to rather decrease excitability [10], [19]. However, also near −80 mV, the discharge in response to the same current step increased in D1 MSNs (n = 14, p = 0.0075), thus mirroring increases in excitability by D1 agonist across membrane potentials in bird area X [20], whereas no change was observed in D2 MSNs (n = 12, p = 0.508, Fig. 4*C, D*). These results show DAergic effects on whole-cell excitability that partly differ depending on membrane voltage and neuron type.

### Excitability Increase in the Direct Pathway is Robust Across Protocols

We subjected the MSNs to a series of current protocols scaled to an initial near-threshold pulse (see **Materials and Methods**). This procedure ensures that step and ramp current amplitudes are changed in relation to input resistance of individual neurons, thus making their effect on the membrane voltage better comparable.

**Figure 5:**
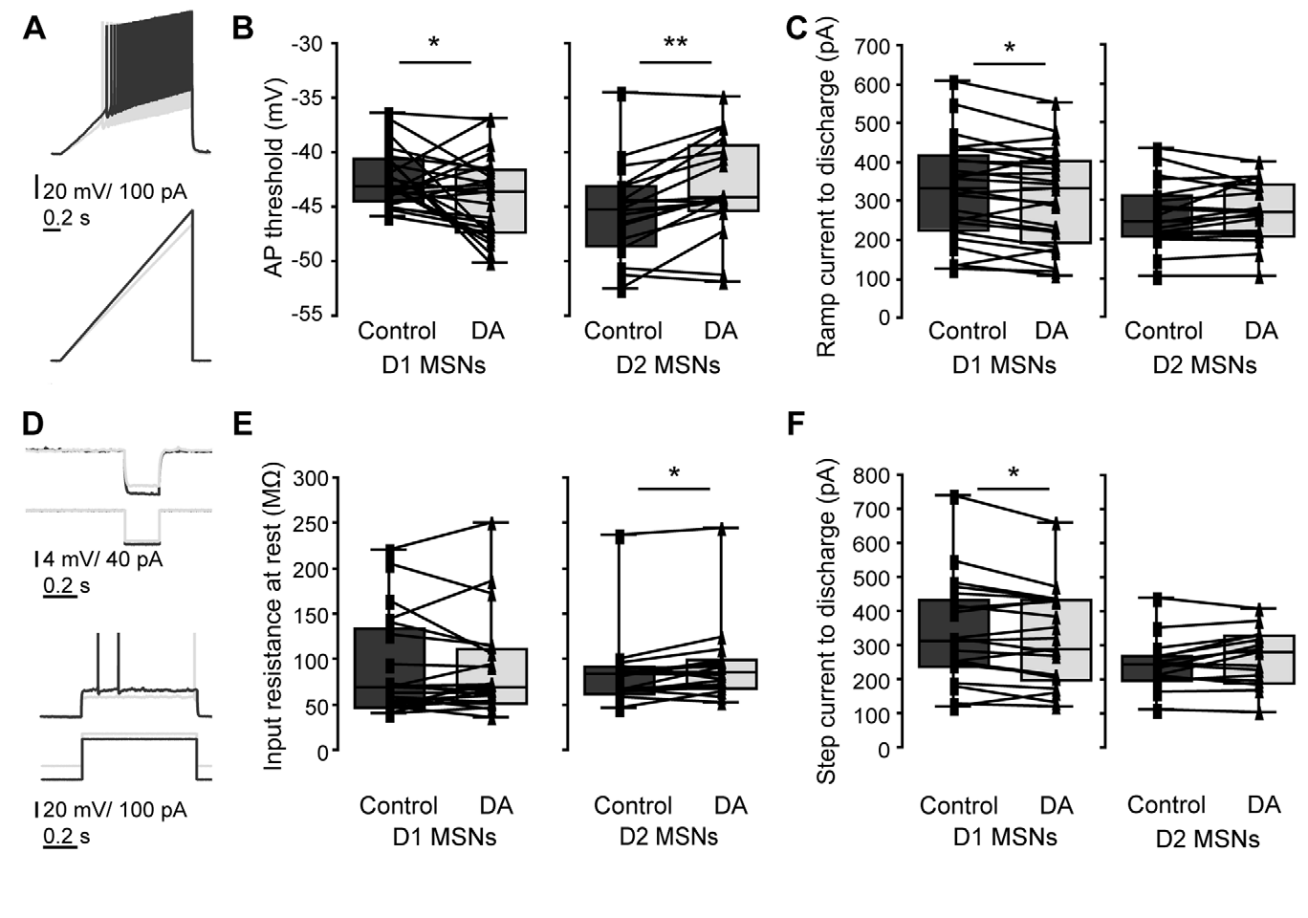
Excitability of D1 and D2 MSNs before and after DA. Excitability of D1 MSNs increases after application of DA (60 µM). *A, B, C*, excitability as measured by ramp current injections in D1 and D2 MSNs. *A,* voltage response and APs of a MSN to ramp current injections before and after DA (dark and light gray). *B,* left panel, box plots showing a decreased AP threshold in D1 MSNs (p = 0.0496). *B,* right panel, box plot: in D2 MSNs, AP threshold was strongly depolarized after DA (p<0.001). *C*, left, box plot comparing minimal currents to discharge in D1 MSNs. Significantly less current was needed following DA application (p = 0.017), thus making D1 MSNs more excitable. Right panel: In D2 MSNs, no significant differences in excitability were observed (p = 0.32). *D*, upper panel, voltage response of MSNs before and after DA (dark and light gray) to small negative step used for extraction of input resistance at baseline membrane potential. Lower panel, voltage responses to minimal suprathreshold current injections. *E,* box plots showing that input resistance measured from rest remained unchanged in D1 MSNs (left, p = 0.392), and increased in D2 MSNs (p = 0.020). *F*, box plots showing changes in the minimal step current that initiates discharge. *F,* left, to bring D1 MSNs to discharge, less current is needed in presence of DA (p = 0.02). *F,*right, in D2 MSNs no significant differences in excitability by step currents were observed (p = 0.32), consistent with the opposing changes in AP threshold and input resistance shown above. For result details see also Tables 3 and 4.

Consistent with the effects on neuronal discharge, DA (60 µM) application increased D1 MSN excitability, as seen in different measures (Fig. 5*B, C, F*). Specifically, the AP threshold of D1 MSNs decreased (p = 0.05, Fig. 5*B*). This was paralleled by smaller ramp and step currents leading to discharge (ramp current: p<0.05; step current: p<0.05, Fig. 5*C, F*). Conversely, DA application increased the AP threshold of D2 MSNs (p<0.001, Fig. 5*B*). However, it also led to an increased input resistance at baseline (p = 0.020, Fig. 5*E*). This resulted in no significant changes in ramp current leading to the first AP (p = 0.322, Fig. 5*C*), and no significantly diminished excitability as measured by step currents (p = 0.061, Fig. 5*F*).

Decrease of excitability in D2 MSNs may be related to the concentration of DA used. In a subset of data, we used higher DA concentrations. Electrical properties of D1 and D2 MSN before and after application of 120 µM DA are summarized in Table 5. The minimal step and ramp currents for threshold discharge were increased in D2 MSNs, and so was the AP threshold, showing a significant decrease in excitability of D2 MSNs (ramp and step current to discharge: p = 0.007 and 0.004, respectively; AP threshold: p>0.0001). Application of higher doses of DA had similar effects on AP properties in D2 MSNs as 60 µM DA, confirming the results described above. Interestingly, the application of high DA concentrations did not increase the excitability of D1 MSNs. Contrary to the effects of lower DA concentrations on D1 MSNs, the threshold for discharge became more depolarized, (n = 5, p = 0.035), and discharge currents were not significantly different (ramp current to first AP p = 0.51; minimal step current to discharge p = 0.26).

**Table 5:** Effect of dopamine (120 µM) on membrane properties of D1 and D2 MSNs.

	D1 MSNs						D2 MSNs					
	Control		DA				Control		DA			
	mean	SD	mean	SD	n	p	mean	SD	mean	SD	n	p
Baseline current (pA)	−4.17	60.03	−77.92	83.20	4	0.093	**−8.88**	87.23	**−88.97**	87.67	10	**0.006**
Baseline membrane potential (mV)	−77.89	1.67	−77.02	2.05	5	0.284	−80.33	3.13	−78.87	4.81	9	0.247
Amplitude first AP (mV)	80.73	5.33	77.80	3.13	5	0.126	74.16	9.05	71.94	7.27	10	0.203
Width first AP (ms)	0.82	0.06	0.87	0.06	5	0.077	**0.81**	0.13	**0.87**	0.12	10	**0.037**
Amplitude second AP (mV)	64.81	10.00	58.01	10.24	4	0.064	**61.78**	6.46	**52.97**	4.60	10	**0.004**
Width second AP (ms)	1.10	0.19	1.13	0.17	4	0.546	1.05	0.19	1.08	0.15	10	0.376
Input resistance rest(MΩ )	79.96	37.56	88.78	39.73	5	0.103	112.80	58.51	102.40	46.01	10	0.219
Time constant for delta pulse (ms)	5.02	1.47	4.51	1.43	5	0.105	**6.47**	3.22	**5.06**	2.99	10	**0.013**
Threshold for APs (mV)	**−44.15**	2.88	**−39.09**	3.25	5	**0.034**	−42.36	4.29	−34.88	4.91	10	**0.000**
Ramp current to first spike (pA)	325.46	142.54	339.13	176.90	5	0.507	**245.90**	95.11	**302.60**	121.90	10	**0.007**
Minimum step current for discharge (pA)	329.98	161.40	366.55	221.63	5	0.255	**251.20**	105.00	**309.10**	128.40	9	**0.004**

These results suggest that the DAergic effect on excitability is stable across stimulation protocols in direct pathway neurons, but that it varies with the concentration of DA used, and that this dependency differs between the two projection systems.

One of the main rationales behind our experiments was to examine effects of DA on excitability of identified MSNs belonging to different (functionally important) pathways. A difficulty with bath applying DA to slices is its oxidation, and comparatively high concentrations are commonly used [20]–[24]. An alternative is to add antioxidants such as ascorbic acid to the bath solution, but this can lead to independent effects on neuronal excitability [25]–[27]. The DA concentrations of 60 µM DA in our experiments are however more representative for the *in vivo* situation [24], [28], [29], and we observed consistent increases of direct pathway excitability at these concentrations. We thus chose to focus on the direct pathway in further experiments.

### Dopamine and D1 Agonist Increase Direct Pathway Excitability during Blockade of Synaptic Transmission

In the slice preparation, much of the striatal microcircuitry is intact, increasing the functional relevance of the DA-mediated effects on excitability of identified MSNs. However, excitability increases in D1 MSNs after application of DA could be mediated directly by the action of DA on intrinsic conductances, or indirectly by affecting the synaptic transmission of connected neurons.

**Figure 6:**
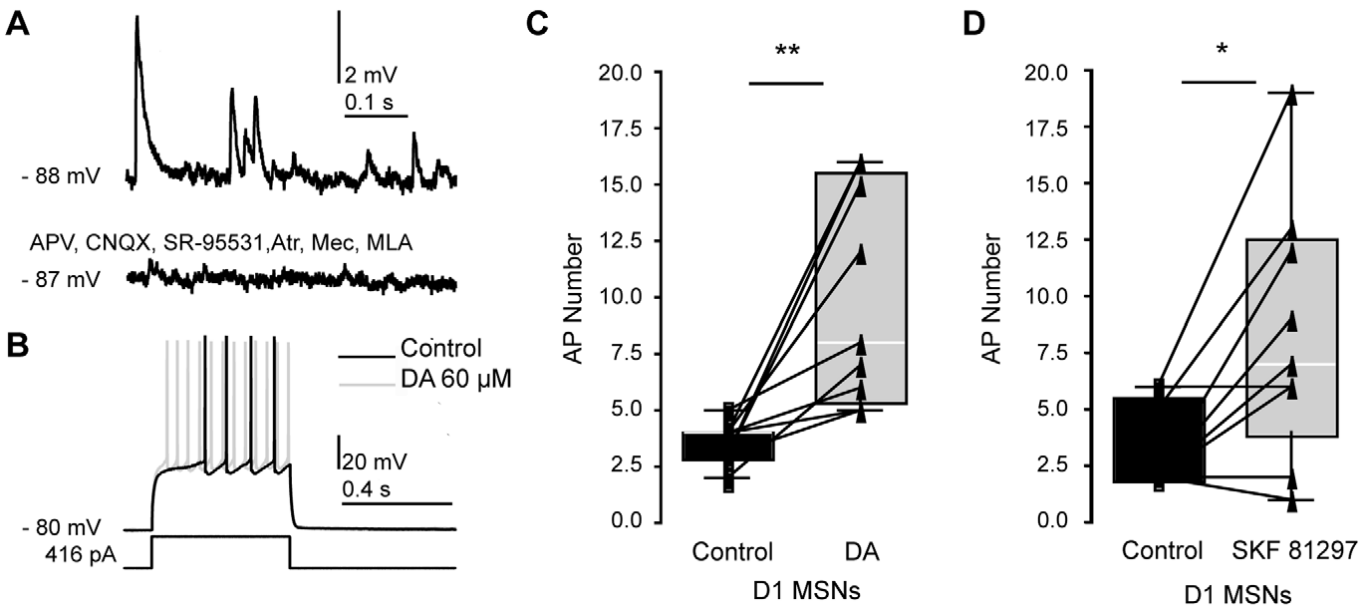
Direct pathway excitability increases when applying DA or D1 agonist during blockade of synaptic transmission. *A*, spontaneous synaptic activity is absent after addition of CNQX (10 µM), AP V (12.5 µM), SR-95531 (10 µM), Atropine (1 µM), MLA (10 nM) and Mecamylamine (10 µM) to the bath. *B*, example response of a D1 MSN to suprathreshold step currents before and after application of DA (dark and light gray traces, respectively), demonstrating positive modulation of discharge also in presence of blockers of synaptic transmission. *C*, effect of DA on excitability in n = 9 D1 MSNs when stimulating from near - 80 mV (−81.00±1.41 mV for control traces and −80.58±1.38 mV after DA application) with drugs present in the bath. Discharge increased significantly (number of APs before DA application: 3.56±0.88, after DA: 10.00±4.73, p = 0.0041, two-tailed paired t-test). *D,* discharge also increased after addition of SKF 81297 (1 µM) to the bath (n = 9 D1 MSNs, number of APs in control: 3.22±1.9, SKF 81297∶8.33±5.7, p = 0.01, with - 81.26±2.0 mV for control traces and - 81.10±2.2 mV after agonist application).

In order to address this question, we investigated the effect of DA on D1 MSN excitability while blocking muscarinic and nicotinergic cholinergic, as well as GABAa and glutamatergic AMPA and NMDA receptor mediated signalling (Fig. 6). Spontaneous synaptic activity was virtually absent after addition of Atropine (1 µM), MLA (10 nM) and Mec (10 µM), as well as GABAzine (10 µM), CNQX (10 µM) and AP5 (12.5 µM) to the bath (Fig. 6*A*), but DA still strongly increased AP discharge (p<0.01, Fig. 6*B, C*).

Nonspecific effects of DA through serotonergic mechanisms are an unlikely reason for increased excitability in D1 MSNs, as *in vivo,* serotonin acts to decrease excitability in striatal neurons [30], [31]. Also via network mechanisms, MSN excitability should be reduced rather than increased based on increased discharge of cholinergic interneurons and positive modulation of FS excitability by serotonin and their respective interconnectivity with striatal MSNs [32]–[35]. We however tested the effect of low micromolar concentration of D1 agonist (SKF 81297, 1 µM) on excitability of D1 MSNs, while blocking muscarinic and nicotinergic cholinergic, as well as GABAa and glutamatergic AMPA and NMDA receptor mediated signaling. Also under this condition, excitability of D1 MSNs increased significantly (n = 9, p = 0.01, Fig. 6*D*). Excitability of D1 MSNs did however not change when stimulating with the same protocol without adding drug to the bath (n = 10 D1 MSNs in control ECS, p = 1.00, data not shown).

The experiments above strongly suggest that the increase in excitability is caused by direct action of DA on direct pathway MSN-intrinsic conductances and not by synaptic modulations. They furthermore show that excitability increases are mediated by D1 receptors.

## Discussion

We investigated intrinsic membrane properties, as well as modulation by DA in projection MSNs of the direct and indirect striatal output pathways, using two model systems. Direct pathway MSNs were identified by retrogradely labeling striatonigral MSNs in rats and by EGFP expression of D1 MSNs in mice. We thus for the first time extensively analyzed electrophysiological parameters of the different MSN types in the rat model and related it to data from transgenic mice. We show excitability differences in both species, with striatonigral direct pathway MSNs being less excitable than their nonlabeled counterparts. We furthermore demonstrate that DA induces depolarization and increases excitability in direct pathway D1 MSNs, and decreases excitability in D2 MSNs. Experiments performed in the presence of synaptic blockers show that increases in excitability in the direct pathway were mediated by direct effects of DA on the recorded MSN rather than indirectly by modulation of network effects and that they were furthermore D1 R mediated. Our results suggest that while the indirect pathway appears more responsive under baseline or low DA conditions, application of DA counteracts this tendency by increasing the intrinsic responsiveness of direct pathway MSNs. These results support classical models of striatal function. Within this scheme, multiple cellular and synaptic mechanisms may interact synergistically to shift the balance between the direct and indirect pathways following DA input.

It is not clear why we did not see an increase in excitability in the direct pathway when applying high DA concentrations. One probable underlying mechanism are differential modulation of sodium currents, as the changes in AP threshold in D1 MSNs were opposite than for the lower DA concentration, and consistent with older literature of DA effects on unselected MSNs using similarly high DA concentrations (see below). Also, the differential impact of DA effects on interneurons (as this subset of experiments was conducted in absence of blockers of synaptic transmission), or increased nonspecific DA effects may play a role.

### Are Baseline Excitability Differences Found In Vivo?

In the rat experiments that are part of this study, we use injection volumes that lead to large areas of striatum being labeled. Furthermore, within the labeled areas, we observed high percentages of labeled cells. Assuming topography of striatonigral projections and random intermingling of direct and indirect pathway neurons, our strategy of selecting nonlabeled neurons near the labeled ones maximizes the probability of patching “indirect pathway” cells (the alternative to retrogradely label the indirect pathway by injecting beads to GPe is not viable, as rat direct pathway neurons send collaterals to the GPe [36], [37], and would therefore also be labeled). Using a comprehensive array of stimulation protocols, we show for the first time excitability differences between direct pathway and nearby (putative) indirect pathway projection neurons in the rat. Similar results were obtained in Drd1a-EGFP mice, corroborating the results of other recent studies [5]–[7]. One remarkable feature of our data that should be noted is that, even if on average excitability differences between the pathways can be found (in both species), the current-frequency plots of individual direct and indirect as well as D1 and D2 MSNs overlap in both species (Figs. 1*D* and 2*B*), implying that *in vitro*, D1 and D2 MSNs are not easily discriminable based on their excitability. However, a recent study gives a hint as to whether excitability differences also exist *in vivo*: In a study of Kravitz and colleagues, baseline differences of excitability between D1 and D2 MSNs were accompanied by a two-fold difference in the baseline discharge rate recorded *in vivo* [38]. These results suggest that the baseline difference in excitability indeed causes a difference in the resting discharge rate of the respective projection pathways.

### Dopamine Modulation of Intrinsic Excitability is Consistent with a Functional Model of the BG

Tonic and phasic DA changes in the striatum have been implicated in multiple functions such as action selection or action motivation, as well as action learning [29], [39]–[42]. According to a classical functional model of the BG, the direct striatofugal pathway facilitates and the indirect pathway inhibits movements, and both are differentially modulated by DA via D1 and D2 Rs [1], [43]. Also this model has received support by the *in vivo* study cited above, in which motor activation and inhibition by D1 and D2 MSNs was seen, respectively [38]. This study, however, showed inconclusive results regarding discharge rate changes after intraperiteonal injections with specific DA agonists.

Release of DA in the striatum following electrical stimulation of the medial forebrain bundle was shown to increase discharge in a subset of neurons, mediated via D1 receptor stimulation, and D1 and D2 antagonists have opposite effects on excitability *in vivo* [44], [45]. Few *in vitro* studies have addressed DA or agonist-related effects directly in the two MSN populations [9], [17], [46], [47]. In D2 MSNs, application of D2 agonist reduces excitability [17], [46]. We extend these findings by demonstrating opposite effects of DA on excitability of the two MSN types in mice. The DA induced membrane depolarization of D1 MSNs acts synergistically with increased excitability, and both should serve to enhance the activity of the direct pathway.

### Modulation of Action Potential Properties by Dopamine

Our results show modulation of MSN AP properties (threshold, width, and amplitude) following DA application. A reduction of peak Na^+^ currents has been observed in dissociated striatonigral MSNs by DA and D1 agonists, which was associated with a negative shift in the voltage-dependence of steady-state inactivation [11]. Our data, however, shows a hyperpolarization of the AP threshold following DA application in D1 MSNs, which contributes to the increased excitability. This apparent discrepancy may be related to the concentrations of agonist or DA used in the respective experiments. In unclassified striatal neurons, AP threshold depolarization has been described in response to high (100 µM) but not low (1 µM) DA concentrations [21]. Consistent with this, our results show AP depolarizations only in D2 MSNs following the application of 60 µM DA, whereas both MSN types showed AP threshold depolarizations for high DA concentrations.

In our study, DA-mediated modulations of AP shape were seen predominantly in D2 MSNs, with decreases of AP amplitudes and significant increases in AP duration. Effects of D2 R activation on sodium currents are not easy to interpret, however, the direction of modulation of the sodium inactivation curve can critically determine whether D2 modulation is excitatory or inhibitory [48]. The net effect of DA on MSN excitability is a result of the modulation of multiple conductances, and in D2 MSNs these may oppose each other, causing only moderate changes in overall excitability.

AP shape has been implicated in graded synaptic transmission at mammalian synapses [49]–[53], suggesting a functional link between AP properties and postsynaptic responses in nearby MSNs as well as BG output nuclei. The impact of DA modulation upon D2 MSN synaptic output may therefore be a result not only of MSN discharge rate, but also of the detailed membrane and AP properties.

### Effects of DA on MSN Excitability Depend on Membrane Potentials Resembling those Found in vivo

Changes in MSN membrane excitability can be caused by multiple alterations of voltage-dependent ion channels [48], [54]. Our results show that DA increases excitability of the direct pathway and decreases excitability of the indirect pathway. The extent or presence of the effects was voltage dependent, as tested at two different holding potentials. While an alternation between depolarized (up state) and hyperpolarized (down state) is seen in anesthetised animals [55]–[57], as well as in unanesthetised animals during slow-wave sleep, during wakefulness the membrane potential follows a unimodal distribution [58]. Baseline membrane potentials near −60 mV in our experiments can be seen as approximating the average membrane potential during spontaneous depolarized states in anesthesia (−54.2±6.9, [56]) or slow wave sleep (−65.4±5.8 mV [58]). The average membrane potential in the awake rat of −69.4±9.6 mV lies in the middle of the two baseline potentials tested in our study. As the AP threshold measured by these authors was however more hyperpolarized than in our study (−51.9±5.0 mV), our baseline potential of –60 mV may in fact be closer to that in the awake animal. The hyperpolarized baseline values in our study on the other hand are more representative for extreme values of membrane polarisation found *in vivo*, regardless of the state of vigilance (−81.7±7.4 mV), or for down states observed under anesthesia (−76.6±9.2 mV [56]). We show that the intrinsic membrane properties of MSNs are strongly modulated at hyperpolarized membrane potential, and also at depolarized values. Further studies are needed to show the respective impact of DA on the intrinsic vs. synaptic properties at different vigilance states *in vivo*.

## Materials and Methods

### Ethical Approval

The experimental procedures were approved by the local ethical committee (Stockholms Norra Djurförsöksetiska Nämd, ethical permits N122 and N426 to GS).

### Retrograde Labeling

Striatonigral MSNs were labeled by stereotactic injection of fluorescent latex microspheres (Lumafluor, Naples, FL, USA) into the SN*r* of juvenile Sprague-Dawley rats (postnatal days (PN) 12–13, Fig. 1*A*, [2]). These beads are transported retrogradely by axons that terminate at the site of injection. The latex beads are nontoxic and very stable [59] with no prominent effect on electrophysiological properties [60], and have been used for similar comparisons [61]–[64]. Specifically, no differences in excitability were apparent when comparing labeled and nonlabeled pyramidal cells of cortical layer 5 [63]. Rats were anesthetized with an intraperitoneal injection of a mixture of Fentanyl (B. Braun, Melsungen, Germany) and Medetomidine (Domitor, Orion Pharma, Finland), diluted in 0.9% saline. The surface of the skull was pierced with a 20G Microlance syringe (Becton Dickinson, NJ, USA), and red microspheres were injected with a Hamilton syringe at a volume of 0.4 µl over 1 minute. Stereotactic coordinates were: 2.2 mm lateral from midline, 1.1 mm anterior to lambda and 6.9 mm below the skull surface. In order to allow diffusion of the solution from the injection site, the syringe was left at the place of injection for at least 5 minutes. Bupivacain (Marcain 2.5 mg/ml, Astra Zeneca, Södertälje, Sweden) was used as local anesthetic before the skin was closed with chirurgical glue (Histoacryl, Braun Aesculap, Tuttlingen, Germany). The analgesic Karprofen (Rimadyl, Pfizer, NY, USA) was administered subcutaneously at 5 mg/kg and the rats awakened with ip injections of a mixture of Atipamezole (Antisedan, Orion Pharma, Espoo, Finland, 1 mg/kg) and Naloxone (0.1 mg/kg), diluted in 0.9% saline. After surgery, the pups were returned to their mother’s cage.

### Transgenic Animals, Slice Preparation and Recordings

Brain slices (thickness 300 µm for rats, and 250 µm for mice) were obtained on PN 14–19, as well as PN 15 or 21–32 for rats and mice, respectively. Slices were parasagittal, except in some rat experiments in which coronal slices were used. As no differences were apparent in intrinsic MSN properties, the data was pooled. Labeled and nonlabeled MSNs were recorded in striatal slices from both retrogradely labeled rats and from bacterial artificial chromosome (BAC) Drd1a-EGFP mice. These transgenic animals express EGFP under control of the promoter for the D1 R (Drd1a) were originally generated by the Gene Expression Nervous System Atlas program at the Rockefeller University [65] and had been crossed on a C57/BL/6 background [66]. Slices were cut in ice-cold extracellular solution, kept at 35°C for 30 minutes, and moved to room temperature before recordings. Whole-cell patch clamp recordings were obtained from striatal MSNs at ca 34–35°C. MSNs were visualized using IR-DIC microscopy (Zeiss Axioskop, Oberkochen, Germany), and retrogradely labeled, as well as EGFP expressing MSNs were identified by switching from infrared to epifluorescence mode. In retrograde labeling experiments, areas with high percentage of labeled MSNs were chosen for recordings. The membrane responses of retrogradely labeled rat MSNs were characteristic for MSNs, with relatively hyperpolarized resting membrane potential (−69.77±10.74 mV), delayed discharge in response to depolarizing current steps, and inward rectification (Fig. 1*B*).

In mice, we used information about D1 R expression to separate the different striatal output pathways. We chose to use D1EGFP expressing cells to identify the direct pathway for several reasons: Previous studies in BAC mice have shown complete colocalization of D1 R and retrogradely labeled SNr-projecting MSNs, as well as no or extremely low (0.7%) colocalization of retrograde labeling and D2 MSNs [5], [18]. Also, the large majority of the nonlabeled MSNs can assumed to be D2 R expressing, as D1 and D2 R expression was mutually exclusive in double transgenic lines [67], and Matamales et al. saw full coverage of DARPP positive neurons by either Drd1a or Drd2-EGFP labeling. Apart from the fact that Drd2-BAC-EGFP mice have been problematic, [68], [69], identifying the direct pathway and comparing to a nonlabeled population makes the two approaches comparable across species. We furthermore chose clearly nonfluorescent cells (with cell bodies appearing darker than background neuropil) to be recorded from. As in the rat model, nonlabeled MSNs were identified by typical membrane properties such as inward rectification from hyperpolarized resting membrane potential, and delayed AP discharge [2].

The extracellular solution both for cutting and recording contained (in mM) 125 NaCl, 25 glucose, 25 NaHCO_3_, 2.5 KCl, 2 CaCl_2_, 1.25 NaH_2_PO_4_, 1 MgCl_2_. Recordings were amplified using multiclamp 700B amplifiers (Molecular Devices, CA, USA), filtered at 2 KHz, digitized (5–20 KHz) using ITC-18 (Instrutech, NY, USA), and acquired using the custom-made routine PulseQ (R. Holzer, unpublished) running on Igor Pro (Wavemetrics, OR, USA). Patch pipettes were pulled with Flamming/Brown micropipette pullers P-97 and P-87 (Sutter Instruments Co, Novato, CA) and had initial resistances 5–10 MΩ. The intracellular solution contained (in mM) 10 HEPES, 0.3 GTP, 10 Na-phosphocreatine and 105 K-Gluconate, 30 KCl, 4 ATP-Mg in mouse experiments. In rat experiments, the above concentrations were modified, using 110 K-Gluconate, 10 KCl, 4 ATPNa and 4 MgCl_2_. Liquid junction potential was not corrected for. Recordings were performed in current clamp mode, with pipette capacitance and access resistance compensated throughout the experiment. Data was discarded when access resistance increased beyond 30 MΩ.

### Stimulation Protocols and Analysis

Passive and active electrophysiological properties were obtained from the membrane responses of MSNs to current injections in current-clamp mode. Recorded MSNs were brought to a similarly hyperpolarized baseline membrane potential (p = 0.731 and p = 0.266 for rat and mouse MSNs of the different types, respectively, Tables 1 and 2). A series of somatic current injection protocols was applied, scaled to an initial near-threshold step pulse designed to capture key active and passive properties [70]. Thus, stimulation of the MSNs was relative to their respective rheobase. More specifically, the subthreshold current-voltage relationship was calculated based on equidistant 1 s step currents (see Fig. 1*B*). We used 50 ms step currents of increasing amplitude (Fig. 1*C*) to extract properties of the first two action potentials (APs). A 1.5 s ramp current (Fig. 1*D*) was used for the extraction of AP threshold and the current-to-first-AP measurement, and the discharge responses to 2.0 s step currents just around threshold (Fig. 1*D*) were used for the calculation of minimal current for threshold discharge and individual current-frequency relationships. A small negative 0.2 s long step current from baseline or on top of a depolarizing or hyperpolarizing step (Fig. 1*F*) was applied to extract input resistances and time constants at different membrane potentials, and a brief (5 ms) hyperpolarizing step current from baseline was used for the extraction of the membrane time constant.

Electrical properties were extracted with IGOR Pro, using a custom-written routine for the characterization of the electrophysiological properties of neurons (R Holzer, unpublished). For single AP analysis, averages of AP properties of typically 6 sweeps were used. APs were detected with fixed threshold slopes of 10 mV/ms in rat MSNs with adaptations up to 22 mV/ms, and slopes of 40 mV/ms for mouse MSNs. The first AP in response to a ramp current injection was detected with a threshold slope of 10 mV/ms. Input resistances were obtained on averages of the membrane potential within a window of 0.09 s before and during the negative current step. We calculated time constants by fitting the decay phase of the membrane potential to this current step or, in case of stimulation with a short hyperpolarizing pulse, the recovery phase from hyperpolarization with a single exponential. Some results of this analysis are highlighted in Figs. 1, 2, 3 and 5. Numerical breakdowns for MSN subtypes in the two model systems and for each subtype before as well as after DA application are displayed in Tables 1 to 5.

Two-tailed, two-sample Students t-tests (preceded by F-tests to evaluate homogeneity of variances) were used for comparison of direct pathway and nonlabeled MSNs in rats and mice (Figs. 1 and 2 and Tables 1 and 2; for mice, only MSNs for which DA modulation data existed were taken into the analysis) and two-tailed paired t-tests for assessing the impact of DA application on multiple membrane properties when stimulating from hyperpolarized membrane potentials (Tables 3, 4, 5; Figs. 3, 5, 6). An ANOVA was computed in Prism (Graph Pad Software Inc.) for comparison of membrane potential changes shortly after DA application. For excitability analysis at depolarized membrane potential (Fig. 4*B*), one-tailed t-tests were used. Data is presented as mean ± SD, and for all figures one star (*) denotes p<0.050 and two stars (**) denote p<0.010. Data presented as box plots is shown with the central bar denoting the median and the box encompassing the interquartile range (extending from the 25^th^ percentile to the 75^th^ percentile); the whiskers expand to the highest and lowest values.

### Drug Application

After recording of baseline active and passive membrane properties at hyperpolarized membrane potentials in mouse MSNs, dopamine hydrochloride (SIGMA-Aldrich, St. Louis, MO) was bath applied in extracellular solution to final concentrations of 60 and 120 µM. The short-term effect of DA on membrane potential and excitability at relatively depolarized or at hyperpolarized membrane potential was observed by repeatedly applying equal-amplitude current steps eliciting AP responses (0.5 s current steps every 30 s, using membrane potential measurements before and 3 minutes after DA application, and adjusting the membrane potential afterwards). In Figures 4 and 6, AP numbers in response to equal amplitude current steps are compared at similar membrane potential (using the trace with the membrane potential closest to control values within a certain time window, and discarding all traces with a baseline membrane potential deviating more than 3 mV from control. For all mouse MSNs included in the detailed analysis of membrane properties, the extensive stimulation protocol was repeated after more than 5 minutes near −80 mV (Figs. 3 and 5, Tables 3 to 5). In experiments depicted in Fig. 6, synaptic signaling was blocked by SR-95531 (GABAzine, 10 µM), Atropine (1 µM, both Sigma-Aldrich), as well as CNQX (10 µM), AP5 (12.5 µM, Methyllycaconitine citrate (MLA, 10 nM) and Mecamylamine hydrochloride (Mec, 10 µM, all Tocris Bioscience) in the bath solution. D1 agonist SKF 81297 (Tocris Bioscience) was bath-applied at 1 µM.
